# Pathogenicity of *Steinernema carpocapsae* ALL Entomopathogenic Nematodes and Their Symbiotic Bacteria as a Biological Control Agent on Red Palm Weevil

**DOI:** 10.3390/microorganisms13050971

**Published:** 2025-04-24

**Authors:** Chaojun Lv, Taigao Meng, Baozhu Zhong, Zhongqiu Shang, Chaoxu Li, Abdullah A. Zahra, Talat M. Abdelrahman

**Affiliations:** 1 Coconut Research Institute, Chinese Academy of Tropical Agricultural Sciences, No. 496 Wenqing Avenue, Wenchang 571339, China; lcj5783@126.com (C.L.); chaoxu998@163.com (C.L.); 2 College of Tropical Crops, Yunnan Agricultural University, Puer 665099, China; mtg2730@163.com (T.M.); shangzhonqiu@163.com (Z.S.); 3 Plant Protection Department, Faculty of Agriculture, Al-Azhar University, Cairo 11884, Egypt; abdullah.zahra@azhar.edu.eg; 4 Plant Protection Department, Faculty of Agriculture, Al-Azhar University, Assiut 71524, Egypt

**Keywords:** *Steinernema carpocapsae* ALL, symbiotic bacterium, *Rhynchophorus ferrugineus*, bio-control, entomopathogenic nematodes

## Abstract

Insect-specific pathogens present a sustainable alternative to pesticides for managing the red palm weevil (RPW). This study assessed the efficacy of *Steinernema carpocapsae* ALL nematodes and their symbiotic bacteria against the third-instar larvae and adults of RPW under laboratory conditions. The symbiotic bacteria were isolated, morphologically characterized, and genetically identified. The results indicated that the mortality rates of RPW larvae treated with *S. carpocapsae* exceeded 50% in all treatments at 120 h, reaching 93.33% at a concentration of 250 IJs/mL. The morphology of isolated symbiotic bacterium from *S. carpocapsae* on NBTA medium exhibited a light green color with a glossy surface, a raised center, and a mucilaginous texture. A novel strain of symbiotic bacterium was identified and named as LZ-G7. The bacteria toxicity on RPW adults showed a notable mortality rate of 66.67% at 48 h after feeding with concentration of 10 × 10^7^ CFU/mL. The mortality rate of the third-instar larvae of RPW reached 83.33% after feeding with 0.30 × 10^8^ CFU/g at 96 h and 93.33% after injection into blood cavity with 8 × 10^6^ CFU at 48 h. These results suggest that *S. carpocapsae* and a novel symbiotic bacterium strain exhibit strong virulence against RPW and have the potential to serve as effective biological control agents in integrated pest management strategies.

## 1. Introduction

For over 30 years, the red palm weevil *(Rhynchophorus ferrugineus, RPW)* has posed a serious threat to palm cultivation worldwide, causing substantial ecological and economic harm in various agricultural regions [1]. This invasive pest targets over 30 palm species, including key crops like date, coconut, and oil palms, using concealed feeding strategies and displaying resilience to environmental challenges [2,3]. Its larvae develop inside the trunk, silently inflicting severe internal damage that often goes unnoticed until the infestation is advanced [2,4,5]. Entry typically occurs through the base of the petiole, open wounds, or the crown’s growing point, making early detection particularly difficult. By the time visible signs such as leaf collapse or wilting appear, the infestation is usually well established and the internal structure significantly compromised [6,7,8,9,10]. Conventional chemical treatments, though effective, raise concerns regarding resistance development, environmental impact, and worker safety [11,12]. In contrast, biological control agents like entomopathogenic fungi and nematodes have emerged as sustainable alternatives. These include species such as *Beauveria bassiana* [13], *Isaria fumosorosea* [14], *Metarhizium anisopliae* [15,16], *Lecanicillium lecanii* [17,18], and *Steinernema carpocapsae* [19,20], offering promising avenues for integrated pest management strategies.

Entomopathogenic nematodes (EPNs), such as *S. carpocapsae*, are notable for their adaptability to diverse environmental conditions [21,22]. Their efficiency as biological control agents stems from their targeted parasitism, potent pathogenic effects on insect hosts, and minimal ecological impact, making them well-suited for integrated pest management (IPM) strategies [23,24]. Several factors influence the life cycle of EPNs, including ambient temperature, the presence of symbiotic bacteria, and the host insect’s immune defenses [25]. Among these, symbiotic bacteria play an especially pivotal role in the nematodes’ development, infectivity, and survival [26,27,28]. During the infection process, EPN larvae carry these bacteria housed in their gut and release them into the host’s body upon successful entry [29]. Entry occurs through natural openings such as the mouth, anus, spiracles, or wounds in the insect’s exoskeleton. Once inside, the symbiotic bacteria rapidly multiply and secrete various virulence factors, including toxins, enzymes, and immune-suppressing compounds. These agents cause septicemia, leading to the host’s death. After consuming the internal contents of the host, the nematodes exit the depleted body in search of new hosts to infect [30,31].

EPNs have been successfully applied against RPW in multiple studies. Both *S. carpocapsae* and *Heterorhabditis bacteriophora* have shown high efficacy across the pest’s developmental stages, achieving mortality rates of 94.68% and 92.68%, respectively, with larvae being the most vulnerable [32]. Follow-up studies demonstrated variable effectiveness among different strains; for instance, *S. carpocapsae* ItSCAO1 caused 83.3% mortality in larvae and 55% in adults, while *H. bacteriophora* ItH-LU1 achieved 36.7% and 30%, respectively [33]. In another investigation, only 9.09% of surveyed soil samples contained native *S. carpocapsae* and *H. indica*, yet these strains resulted in ~90% larval mortality within 10 days. Higher concentrations (1 × 10^6^ IJs/mL) further enhanced efficacy, and differences in bacterial strains were found to influence pathogenicity [34]. This study aims to evaluate the pathogenicity of the *S. carpocapsae* ALL strain and characterize their associated symbiotic bacterium. The mortality rates of third-instar RPW larvae exposed to different concentrations of *S. carpocapsae* infective juveniles (IJs) were evaluated. The morphology of the isolated symbiotic bacterium from *S. carpocapsae* was examined and characterized. Additionally, the effectiveness of the isolated bacterial strain was tested against RPW adults and third-instar larvae through feeding and injection assays.

## 2. Materials and Methods

### 2.1. Rearing and Propagation of Red Palm Weevil and Entomopathogenic Nematodes

The RPW was reared in the Biological Control Laboratory of the Coconut Research Institute of the Chinese Academy of Tropical Agricultural Sciences (CRI-CATAS). To rear RPW, an artificial diet was prepared by thoroughly mixing ground coconut tissue with wheat flour in a 9:1 ratio. Distilled water was added gradually to achieve a moist consistency suitable for larval development. Adult weevils (20 individuals; 10 males and 10 females) were introduced into a rearing box measuring 30 × 50 cm. Coconut husks were placed on top of the artificial diet to provide a natural substrate for oviposition. The box was covered with a lid perforated with small ventilation holes to allow air exchange while preventing insect escape. The rearing environment was maintained at 27 ± 1 °C, relative humidity ≥70%, and a photoperiod of 16:8 h (light:dark) until egg laying and larval emergence occurred. Only healthy third-instar larvae and adults were selected for use as test insects. The EPNs (*S. carpocapsae* ALL) were propagated by white trap method [35], using *Galleria mellonella* as host. The collected nematodes were adjusted to a final concentration of 1.0 × 10^3^ IJs/mL with distilled water and stored at 8~10 °C for reserve.

### 2.2. Evaluation of Steinernema carpocapsae on the Third-Instar of RPW Larvae

A 150 g artificial diet was prepared and placed in an insect box. Third-instar RPW larvae were selected, and 1 mL of nematode suspensions (50, 100, 150, 200, or 250 IJs/mL) was applied to the thorax of each larva by dripping 200 μL onto the thorax five times, with each application spaced 5 min apart. Larval mortality was recorded at 24, 48, 72, 96, and 120 h post-treatment, and the corrected mortality (LC_50_ and LT_50_ values) was calculated. Each treatment included three replicates, and each replicate consisted of 10 insects, totaling 30 insects per treatment. Sterile water was used as the control. The experiment was conducted at 27 ± 1 °C with a relative humidity of 75 ± 3%.

### 2.3. Preparation of Culture Medium of Nematode Symbiotic Bacteria

The components of the media used for culturing the symbiotic bacteria are listed in Table 1. The IJs of *S. carpocapsae* were rinsed with sterile PBS buffer, and the concentration was adjusted to 300 IJs/mL to infect the larvae of *G. mellonella*. After 48 h, the infected and dead *G. mellonella* were selected to cut off the forefoot with disinfection scissors. The double-tube centrifugation method was used to collect the hemolymph [36].

Several small holes were placed at the bottom of a 0.5 mL sterile conical centrifuge tube, and the larvae of the *G. mellonella* with the forefoot cut off were inserted into a 1.5 mL micro-sterile centrifuge tube. After centrifugation with 10,000 rpm and 60 s, the hemolymph was collected from the bottom of the 1.5 mL centrifuge tube. The collected hemolymph was smeared on NBTA medium and placed in a dark constant-temperature incubator at 25 °C. After 36–48 h, the blue-green colonies were picked and cultured in the new NBTA medium to obtain the required nematode symbiotic bacteria. Purified blue-green single colonies were selected and cultured in NA medium at 28 °C with shaking at 170 rpm for 24–48 h. The solution was transferred into a flask containing fresh culture medium at a 10% (*v*/*v*) inoculation rate and incubated with shaking at 180 rpm and 28 °C for 24–48 h. The resulting fermented bacterial liquid was stored at 4 °C.

### 2.4. Identification of Nematode Symbiotic Bacteria

A total of 4 mL of the fermented bacterial liquid was taken to extract DNA according to the protocol outlined in the bacterial genomic DNA extraction kit (Tiangen Biochemical Technology Co., Ltd., Beijing, China). The DNA integrity was detected by 1.5% agarose gel electrophoresis and the purity and concentration of DNA were estimated by QuawellQ5000 ultramicro UV-visible spectrophotometer. The 16S rRNA gene was amplified by PCR. The PCR reaction system and the standard PCR amplification procedure are shown in Table 2. A total of 10 μL of the PCR product was separated using 1.5% agarose gel electrophoresis at 120 V for 10 min. The recovery results were detected using 1.5% agarose gel electrophoresis, following the instructions provided with agarose gel DNA recovery kit (Tiangen Biochemical Technology Co., Ltd.) [37,38].

The purified DNA fragment was ligated with a pMD18-T vector (Takara Bio Inc., Shiga, Japan). The ligation products were shaken in an ice bath in 100 μL DH5α competent cells and placed on ice for 30 min. After 90 s heat shock in a water bath (42 °C), the samples were quickly cooled on ice for 5 min. The LB liquid medium was mixed and cultured at 37 °C and 200 rmp for 1 h. The samples were centrifuged to remove the supernatant, and the remaining liquid was mixed. Then, 100 µL was coated onto LB plates containing Amp+ (100 µg/ mL) and incubated at 37 ± 1 °C for 24 h. The bacterial solution was sent for sequencing, and the sequencing results were compared with the known bacterial 16S rRNA sequences in the National Center for Biotechnology Information (NCBI) database. The phylogenetic tree was drawn to establish its phylogenetic relationship and taxonomic status [39].

### 2.5. Toxicity of Nematode Symbiotic Bacteria Against RPW Adult Through Feeding

The experiment referred to the method of Abdelsalam et al. [40]. The symbiotic bacterial solution was diluted with 10% honey water to concentrations of 0.01 × 10^7^ CFU/mL, 0.1 × 10^7^ CFU/mL, 1 × 10^7^ CFU/mL, 5 × 10^7^ CFU/mL, and 10 × 10^7^ CFU/mL, respectively. The bacterial solution was placed in a 0.5 mL centrifuge tube, and a hole was made in the cap such that its pore size allowed the mouthparts of the RPW to pass through. The mouth of the adult was inserted into the tube through the hole, and the insect body was fixed with a sealing film. Each treatment of 10 adults was repeated three times, with the same volume of 10% honey water treatment as a control. The mortality rates of adults were calculated at 12, 24, 36, and 48 h after treatment.

### 2.6. Toxicity of Symbiotic Bacteria on the Third-Instar RPW Larvae via the Feeding

The fermentation broth was mixed with artificial feed at a final concentration of 0.10 × 10^8^ CFU/g, 0.15 × 10^8^ CFU/g, 0.20 × 10^8^ CFU/g, 0.25 × 10^8^ CFU/g, and 0.30 × 10^8^ CFU/g, respectively. A total of 10 g of mixed feed was placed in a rearing cup (φ5 × 7 cm), and a third-instar larvae of red palm weevil washed with sterile water was placed. The mortality of the test insects was examined, and the corrected mortality was calculated at 24, 48, 72, 96, and 120 h after treatment. Each treatment was repeated three times with 10 test insects, and the artificial feed treatment without fermentation broth was used as the control.

### 2.7. Toxicity of Symbiotic Bacteria on the Third-Instar RPW Larvae via the Injection

To evaluate the toxicity of symbiotic bacteria using the injection method [41], the symbiotic bacterial concentration was adjusted to 0.5 × 10^8^ CFU/mL, 2 × 10^8^ CFU/mL, 4 × 10^8^ CFU/mL, 6 × 10^8^ CFU/mL, and 8 × 10^8^ CFU/mL with PBS, and 10 μL of each diluted concentration was injected into the blood cavity of the larvae from the left anterior limb of third-instar larvae of RPW with a capillary needle (amount of injected bacteria in each treatment was 0.5 × 10^6^ CFU, 2.0 × 10^6^ CFU, 4.0 × 10^6^ CFU, 6.0 × 10^6^ CFU, and 8.0 × 10^6^ CFU, respectively). The death of the tested insects was checked 48 h after treatment and the corrected mortality was calculated. Each treatment contained 10 test insects and was repeated three times with an injection of PBS as a control.

### 2.8. Data Analysis

Experimental data were processed using Excel 2017 to calculate means and standard errors (mean ± SE). Virulence data were analyzed following the methodology outlined in reference [42], including the calculation of mortality rates, virulence coefficients, and their 95% confidence intervals. The LC_50_, LD_50_, and LT_50_ values were estimated through probit analysis using SPSS version 20.0 (SPSS Inc., Chicago, IL, USA), based on mortality data collected from bioassays at varying concentrations and exposure times.

## 3. Results

### 3.1. The Pathogenicity Evaluation of S. carpocapsae on the Third-Instar RPW Larvae

**Figure 1:**
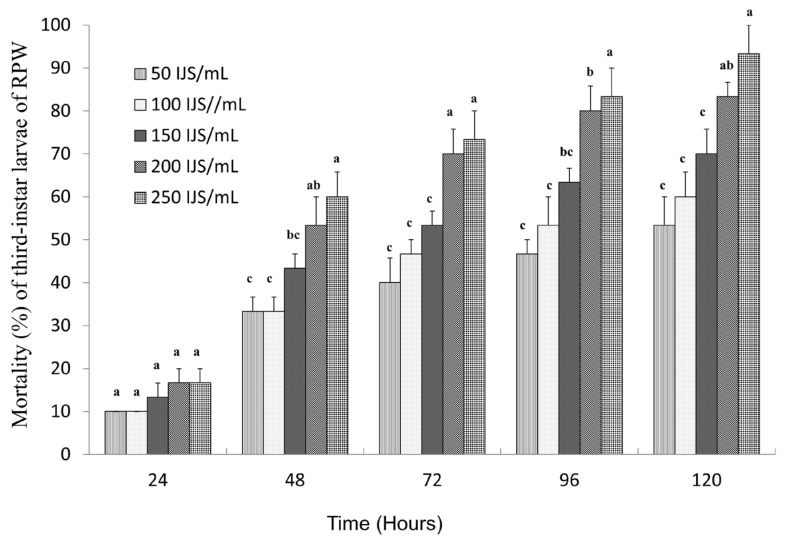
Mortality percentage of third-instar larvae of RPW after treatment with *S. carpocapsae.* The letters (a, b, c, etc.) indicate statistically significant differences (*p* < 0.05) among treatments on the same day.

The pathogenicity of *S. carpocapsae* nematodes against third-instar RPW larvae was evaluated at different concentrations. The results demonstrated a clear concentration and time-dependent increase in larval mortality (Figure 1). At 24 h of treatment, the mortality rates remained below 20% across all tested concentrations. However, as the exposure time increased, mortality rates rose significantly, surpassing 50% at 120 h for all concentrations. Notably, the highest dose (250 IJs/mL) achieved a peak mortality rate of 93.33%, confirming the strong virulence of *S. carpocapsae* at higher concentrations. Virulence analysis further revealed a progressive decline in LC_50_ values over time, indicating increased nematode efficacy with prolonged exposure (Table 3). The LC_50_ values gradually decreased from 1139 IJs/mL at 24 h to 57.67 IJs/mL at 120 h, indicating increased nematode efficacy over time. Additionally, higher nematode concentrations significantly reduced the time required to reach 50% mortality (LT_50_), demonstrating a strong dose-dependent effect (Table 3). The LT_50_ values were 96.99, 84.31, 66.81, 49.29, and 45.03 h with the concentrations of 50, 100, 150, 200, and 250 IJs/mL treatments, respectively, indicating a strong dose-dependent effect. This trend highlights the enhanced pathogenicity of *S. carpocapsae* at higher concentrations and its potential for effective biological control of RPW larvae.

### 3.2. Morphological Characteristics of Symbiotic Bacteria Strains from Steinernema carpocapsae ALL

**Figure 2:**
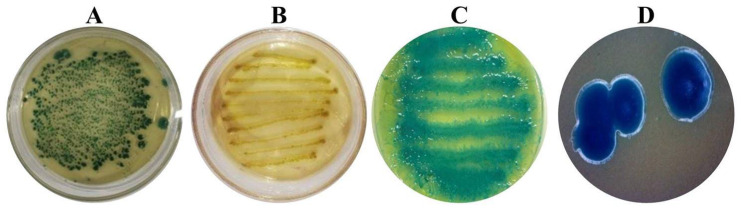
Morphological characteristics of symbiotic bacteria LZ-G7: (**A**) single colony obtained by culturing the hemolymph of infected *G. mellonella* by *S. carpocapsae* on NBTA medium for 48 h, (**B**) morphology of the colony of the initially isolated symbiotic bacteria cultured on the LB medium plate for 48 h, (**C**) morphology of the initial colony for isolated symbiotic bacteria cultured on NBTA medium plate for 48 h, and (**D**) isolated symbiotic cells from NBTA medium.

After Galleria mellonella hemolymph was infected by nematodes and cultured on NBTA identification medium for 48 h, irregular single colonies appeared on the surface of the medium (Figure 2A). The isolated colony was then streaked onto LB medium, where it developed a light yellow, purulent appearance after 48 h (Figure 2B). When streaked onto an NBTA medium plate, the colony absorbed bromothymol blue, forming a distinctive blue-green coloration after 48 h. The colonies on NBTA medium exhibited a shiny surface, a convex central elevation, and a moist, sticky texture when picked up with an inoculation needle (Figure 2C). Symbiotic cells isolated from NBTA medium showed smooth and glossy edges (Figure 2D).

### 3.3. Taxonomic Identification of Symbiotic Bacteria

**Figure 3:**
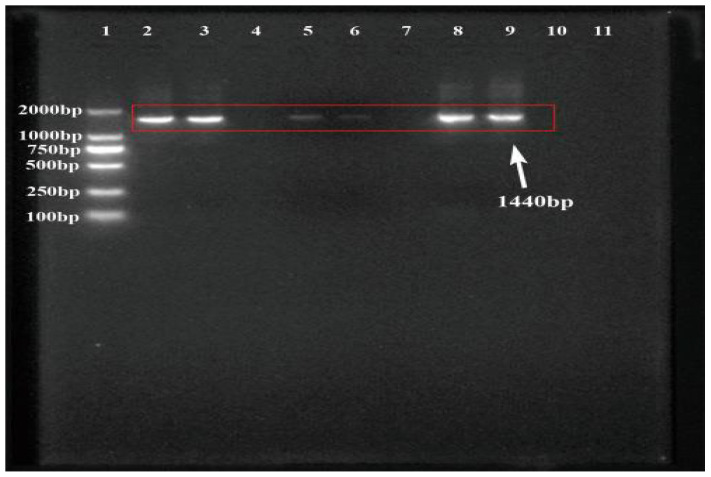
Agarose gel electrophoresis results of PCR amplification products of 16S rRNA of LZ-G7 strain (1: 2000 bp maker; 2, 3: PCR amplification products; 5, 6: DNA recovery products; 8, 9: conversion connection product).

**Figure 4:**
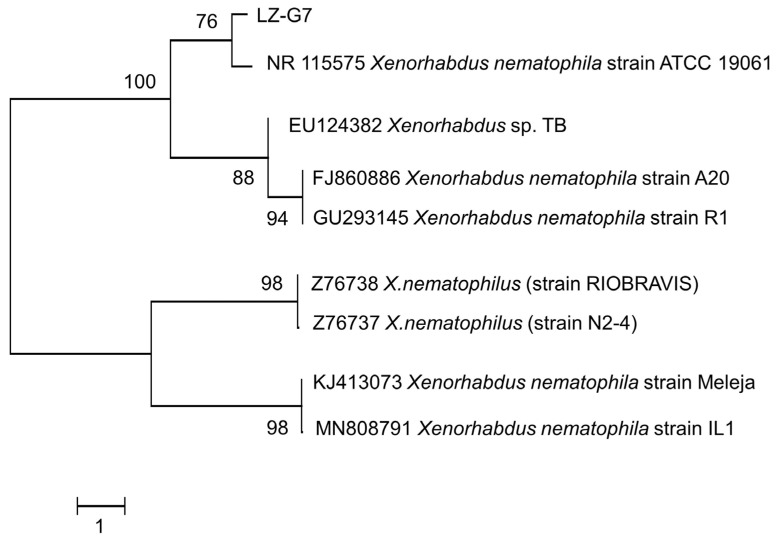
Phylogenetic tree based on 16S rRNA sequences homology.

The sequencing results revealed that the 16S rDNA sequence of the symbiotic strain LZ-G7 was 1440 bp (Figure 3). NCBI alignment showed that the sequence had over 99% similarity with *Xenorhabdus nematophila* strains TB, A20, and R1. The phylogenetic tree was constructed based on the 16 S rRNA sequence homology (Figure 4), and the isolated symbiotic bacteria had the highest homology with *Xenorhabdus nematophila* ATCC19061 from genus *Xenorhabdus*, forming a cluster. The strain LZ-G7 was identified as a novel symbiotic bacterial strain of *S. carpocapsae*, and classified as *Xenorhabdus nematophila*, belonging to the phylum of Proteobacteria, class of γ-proteobacteria, order of Enterobacteriales, and family of Enterobacteriaceae. The NCBI database accession number for this strain is OR150324.

### 3.4. Toxicity of Strain LZ-G7 Following Ingestion by Adult RPW

**Figure 5:**
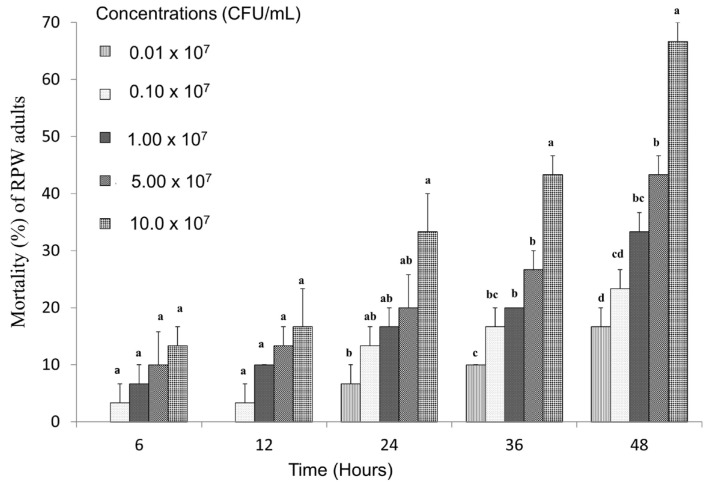
Mortality percentage of RPW adults after feeding with different concentration of LZ-G7. The letters (a, b, c, etc.) indicate statistically significant differences (*p* < 0.05) among treatments on the same day.

The toxicity of nematode symbiotic bacteria at various concentrations against RPW adults through feeding was evaluated, as depicted in Figure 5. The results demonstrated a clear positive correlation between bacterial concentration and the corrected mortality rate of RPW adults, with mortality rates rising as the bacterial concentration increased, indicating concentration-dependent toxicity. At the highest concentration (10 × 10^7^ CFU/mL), the mortality rate reached 13.33% after 6 h of exposure. This rate significantly increased over time, reaching 66.67% after 48 h, suggesting a prolonged and cumulative toxic effect. At the lowest concentration (0.01 × 10^7^ CFU/mL), mortality rates were 6.67% after 24 h and gradually increased to 16.67% after 48 h.

Furthermore, the LC_50_ values for RPW adults at 6, 12, 24, 36, and 48 h were 185 × 10^9^ CFU/mL, 18 × 10^9^ CFU/mL, 9.95 × 10^9^ CFU/mL, 1.7 × 10^9^ CFU/mL, and 0.0457 × 10^9^ CFU/mL, respectively. Moreover, the LT_50_ values were 37.45, 103.27, 166.52, 186.13, and 181.29 h for bacterial concentrations of 10 × 10^7^ CFU/mL, 5 × 10^7^ CFU/mL, 1 × 10^7^ CFU/mL, 0.1 × 10^7^ CFU/mL, and 0.01 × 10^7^ CFU/mL, respectively, as shown in Table 4. These results indicate a slower onset of mortality at lower concentrations, which corresponds with the reduced efficacy observed in the early stages. The gradual increase in mortality over time across all concentrations suggests that the nematode-symbiotic bacteria exert a cumulative toxic effect on RPW adults, likely due to the slow colonization of the digestive system of insects and the gradual release of bacterial toxins.

### 3.5. Toxicity of Strain LZ-G7 Following Ingestion by Larvae of RPW

**Figure 6:**
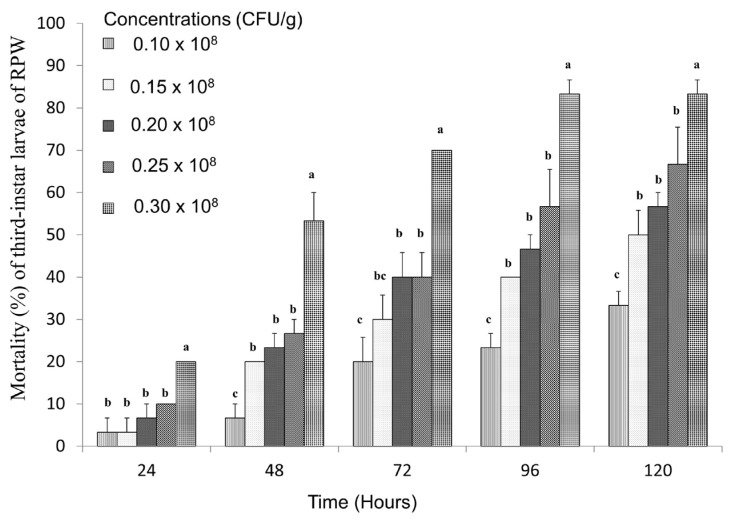
Mortality of RPW third-instar larvae after feeding with different concentrations of LZ-G7 bacteria solution. The letters (a, b, c, etc.) indicate statistically significant differences (*p* < 0.05) among treatments on the same day.

The effect of different concentrations of the LZ-G7 bacterial strain on the mortality of third-instar RPW larvae was evaluated. The results indicated a concentration and time-dependent increase in mortality, with higher bacterial concentrations and longer exposure leading to progressively higher mortality rates. Specifically, after 96 h of treatment, the mortality rate reached 83.33% at the highest concentration (0.30 × 10^8^ CFU/g), while the lowest concentration (0.10 × 10^8^ CFU/g) resulted in a mortality rate of 23.33% (Figure 6). The LC_50_ values demonstrated that the toxicity of the LZ-G7 strain against RPW larvae increased over time. The LC_50_ values decreased progressively with the duration of the treatment, from 0.99 × 10^8^ CFU/g at 24 h to 0.15 × 10^8^ CFU/g at 120 h (Table 5), indicating a cumulative toxic effect. Furthermore, as the bacterial concentration increased, the LT_50_ values also decreased in a similar pattern. The LT_50_ values were 206.77, 116.37, 100.84, 83.96, and 48.48 h for the concentrations of 0.10 × 10^8^ CFU/g, 0.15 × 10^8^ CFU/g, 0.20 × 10^8^ CFU/g, 0.25 × 10^8^ CFU/g, and 0.30 × 10^8^ CFU/g, respectively. This direction suggests that higher concentrations of LZ-G7 not only result in faster mortality but also indicate that the efficacy of the strain is enhanced both by concentration and by longer exposure periods.

### 3.6. Toxicity of LZ-G7 Strain After Injection into RPW Larvae at the Third-Instar

**Figure 7:**
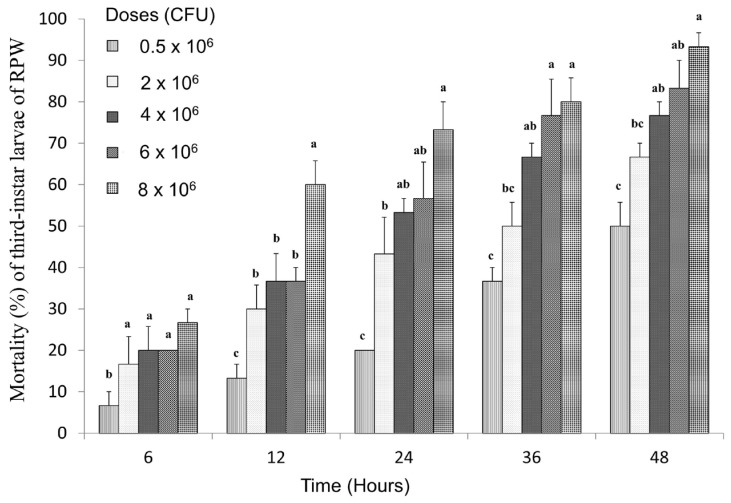
Mortality of the third-instar larvae of RPW after injected with LZ-G7 bacteria solution. The letters (a, b, c, etc.) indicate statistically significant differences (*p* < 0.05) among treatments on the same day.

After injecting LZ-G7 bacterial solutions with different concentrations into the blood cavity of RPW third-instar larvae, a distinct dose- and time-dependent increase in mortality was observed (Figure 7). Mortality rates rose progressively with increasing bacterial concentrations and longer treatment durations. At 48 h of treatment, all tested concentrations resulted in mortality rates exceeding 50%. Specifically, mortality rates at 48 h were 50%, 66.67%, 76.67%, 83.33%, and 93.33% for the doses of 0.5 × 10^6^, 2.0 × 10^6^, 4.0 × 10^6^, 6.0 × 10^6^, and 8.0 × 10^6^ CFU, respectively. Moreover, mortality rates at 6 h of treatment were considerably lower, recorded at 6.67%, 16.67%, 20%, 20%, and 26.67% for the same concentrations. Virulence analysis further confirmed the efficiency of LZ-G7, as the LD_50_ values showed a decreasing trend over time, indicating increased efficacy with prolonged exposure. The LD_50_ values were 76.58 × 10^6^, 7.77 × 10^6^, 3.03 × 10^6^, 1.34 × 10^6^, and 0.63 × 10^6^ CFU after 6, 12, 24, 36, and 48 h, respectively (Table 6). Additionally, the lethal time required to achieve 50% mortality decreased as bacterial concentrations increased. The LT_50_ values were 59.29, 29.37, 19.41, 16.89, and 11.10 h for the doses of 0.5 × 10^6^, 2.0 × 10^6^, 4.0 × 10^6^, 6.0 × 10^6^, and 8.0 × 10^6^ CFU, respectively. This trend indicates that higher bacterial doses result in faster mortality, reinforcing the effectiveness of LZ-G7 in controlling RPW larvae through direct injection. These findings emphasize the crucial role of bacterial dosage and exposure duration in maximizing mortality rates and overall efficacy. Furthermore, they highlight the potential for optimizing bacterial delivery methods to enhance control strategies against the RPW.

## 4. Discussion

The results demonstrated a high pathogenicity of the nematode-bacteria complex against both the third-instar RPW larvae and adults, with efficacy positively correlating with increased concentration and exposure duration. The highest mortality rates were recorded at the highest tested concentrations, indicating a dose- or concentration-dependent response. This suggests that optimizing application rates could maximize effectiveness in field conditions. Furthermore, genetic analysis revealed a novel strain of the symbiotic bacteria, identified as LZ-G7, through 16S rRNA gene sequencing. This strain exhibited distinct genetic variations from previously identified symbiotic bacteria, highlighting its potential as a novel and effective biocontrol agent. Further characterization of its virulence factors could provide insights into its mechanism of action against RPW. Previous studies have consistently demonstrated the effectiveness of EPNs against various insect pests, supporting the findings of our research. For instance, *S. carpocapsae* exhibited corrected mortality rates of 67.50% and 72.36% against *Aethina tumida* (Coleoptera: Nitidulidae) larvae after 4 and 12 days, respectively [43], while another study reported mortality rates of 65.0% and 93.8% against *Batocera lineolata* (Coleoptera: Cerambycidae) eggs and larvae at 4000 IJs/Ml [44]. These results underscore the broad-spectrum pathogenicity of *S. carpocapsae*, which aligns with our observed 93.33% mortality in RPW larvae after 120 h at 250 IJs/mL. In studies targeting *R. ferrugineus*, *S. carpocapsae* and *Heterorhabditis bacteriophora* were consistently among the most effective species. For example, in one study, *S. carpocapsae* caused 100% mortality in sixth-instar RPW larvae within 240 h and 83.60% mortality under field conditions [32]. Our results closely parallel these findings, particularly in the larval stage, with high virulence observed even at lower concentrations, as evidenced by our LC_50_ of 57.67 IJs/mL and LT_50_ of 45.03 h. These metrics indicate a more rapid and efficient killing potential compared to studies using higher concentrations or longer exposure times. Other reports have shown variability in EPN efficacy across life stages, with significantly lower mortality in RPW adults and pupae [32,45]. Our study focused primarily on larval stages, which are known to be more susceptible, and our results reaffirm the preference for targeting early instars for optimal control. Furthermore, comparisons with indigenous and commercial EPNs revealed that *H. indica* and *S. carpocapsae* achieved up to 90% mortality in multiple RPW stages within 5–10 days [34], highlighting their strong potential, albeit with varying field persistence. In broader applications, EPNs such as *S. glaseri*, *S. abbasi*, and *S. scapterisci* demonstrated high virulence against adult RPWs and other pests, with some strains achieving 100% mortality at higher inoculum levels [46,47]. However, our results emphasize the efficiency of the *S. carpocapsae* ALL strain in achieving high mortality at relatively low concentrations, suggesting a cost-effective alternative for integrated pest management (IPM). Although *S. carpocapsae* ALL demonstrated high virulence against RPW under laboratory conditions, extrapolating these results to field conditions must be approached with caution. Laboratory environments offer tightly controlled conditions with consistent temperature, humidity, and direct nematode–host interaction, allowing for reproducible and accurate assessments. In contrast, field environments are far more variable, with fluctuating temperatures, humidity, UV exposure, and microbial interactions that can affect nematode survival and infectivity. Moreover, delivery challenges such as reaching hidden RPW larvae inside palm trunks and the variability in host life stages further complicate field performance. Therefore, future research should prioritize field trials to validate laboratory findings, assess nematode persistence, and optimize application strategies. Integrating *S. carpocapsae* with other biological control agents or protective formulations could enhance field efficacy and support its inclusion in sustainable IPM strategies. Furthermore, previous studies have demonstrated the significant toxic effects of symbiotic bacteria associated with EPNs against various insect pests, supporting the rationale behind exploring these bacteria as biocontrol agents. For instance, the symbiotic bacterium N-Yz1, isolated from *S. carpocapsae*, caused 55% and 83.33% mortality in *Spodoptera frugiperda* larvae within 24 h when injected at doses of 0.1 µL and 0.2 µL of 1.0 × 10^6^ CFU/mL, respectively, while oral administration proved ineffective [41]. This contrast between injection and ingestion underscores the critical importance of delivery method, a finding further supported by our study. Similarly, previous work exploring the interactions among *S. carpocapsae*, its symbiotic bacterium *Xenorhabdus nematophila*, and RPW larvae revealed that live bacteria and nematodes suppress host immune responses by downregulating antimicrobial peptide production, whereas dead cells did not elicit this effect [19]. These results provide a mechanistic basis for the enhanced virulence observed when live symbionts are directly introduced into the insect hemocoel. In another comparative study, RPWs in developmental stages were infected with an antibiotic-resistant *E. coli* strain and two EPN strains (*S. carpocapsae* ItS-CAO1 and *H. bacteriophora* ItH-LU1). Larvae displayed higher resistance to bacterial infection than adults and released fewer EPN progeny despite elevated mortality [33]. Our study contrasts with this finding by demonstrating that *X. nematophila* LZ-G7, when injected at optimized doses, effectively overcomes this larval resistance, suggesting that strain selection and delivery optimization can significantly influence biocontrol outcomes. Additionally, earlier work identified bacterial isolates such as *Bacillus thuringiensis*, *Serratia marcescens*, and *Klebsiella pneumoniae* from RPW larvae, with *B. thuringiensis* achieving 100% mortality at 1 × 10^8^ CFU/mL [48]. While previous research has demonstrated the effectiveness of various entomopathogenic bacteria, our study identifies *X. nematophila* LZ-G7 as a potent alternative, distinguished by its natural co-evolution with EPNs. This relationship may enhance its ecological compatibility, host specificity, and overall effectiveness as a biocontrol agent. Our findings emphasize that the method of delivery, particularly the ability to ensure that sufficient doses of bacterial agents reach the internal tissues of the host, is critical to treatment success. However, delivering these bacteria to RPW larvae, which remain concealed deep within palm tissues, presents a significant challenge. Conventional approaches such as direct injection or surface application are impractical under field conditions. To overcome this barrier, we propose the encapsulation of bacterial extracts within nanomaterials, allowing for targeted and controlled delivery through plant tissues. This strategy holds promise for improving both the efficacy and environmental safety of bacterial treatments and represents a forward-looking integration of nanotechnology into biological pest control. In contrast to chemical insecticides, which carry risks of resistance development and environmental harm, the use of EPNs and their symbiotic bacteria offers a sustainable and eco-friendly alternative. Future research should focus on optimizing nanoparticle formulations, ensuring the stability and virulence of bacterial agents under diverse environmental conditions, and exploring synergistic effects with other biological control methods to enhance the integrated management of RPW.

## 5. Conclusions

This study reports the first discovery of a novel bacterial strain LZ-G7, symbiotically associated with *S. carpocapsae* ALL, and its significant virulence against RPW. The findings highlight the strong potential of *S. carpocapsae* and their symbiotic bacteria as effective biological control agents, demonstrating a high mortality rate of 93.33% at a dose of 250 IJs/mL. Additionally, *Xenorhabdus nematophila* (LZ-G7) exhibited notable virulence against both RPW larvae and RPW adults. The study underscores the crucial role of nematode dose and treatment time in efficacy, emphasizing the importance of precise delivery. Future research should focus on field applications, environmental factors influencing EPN performance, and potential synergies with other biological control strategies. Moreover, optimizing delivery mechanisms for targeting pests is also crucial. Large-scale trials are necessary to evaluate the long-term sustainability and economic feasibility of EPN-based RPW management. Overall, *S. carpocapsae* and its symbiotic LZ-G7 strain present a promising and sustainable approach for controlling RPW infestations in agriculture.

## Figures and Tables

**Table 1:** The components of the media used for culturing the symbiotic bacteria.

LB Culture Medium		NBTA Culture Medium		NA Culture Medium		M Culture Medium	
Component	Content	Component	Content	Component	Content	Component	Content
Tryptone	10.0 g/L	Beef extract	5.0 g/L	Beef extract	5.0 g/L	Glucose	6.0 g/L
Yeast Extract	5.0 g/L	Peptone	10.0 g/L	Peptone	10.0 g/L	Peptone	20.0 g/L
NaCl	10.0 g/L	NaCl	5.0 g/L	Dd H_2_O	1.0 L	MgSO_4_	1.4 g/L
Agar	18.0 g/L	Agar	18.0 g/L	pH	7.0	KH_2_PO_4_	0.72 g/L
dd H_2_O	1.0 L	Bromothymol Blue	0.025 g/L			K_2_HPO_4_	0.98 g/L
pH	7.0	Red tetrazolium	0.04 g/L			dd H_2_O	1.0 L
		dd H_2_O	1.0 L			pH	7.0
		pH	7.0				

**Table 2:** The reaction condition of PCR.

Reaction System (50 μL)		Amplification Procedure			
Regent	Amounts (μL)	Steps	Temperature	Time	Cycle
DNA polymerase 2xTaq PCR MasterMixII	25	pre-denaturation	94 °C	5 min	-
Upstream primer	1	Denaturation	94 °C	30 s	35
Downstream primer	1	Annealing	55 °C	30 s	35
DNA templates	4	Extension	72 °C	90 s	35
ddH_2_O	19	Final extension	72 °C	10 min	-
		Store	4 °C	Forever	-

**Table 3:** Pathogenicity of *S. carpocapsae* on RPW larvae at different exposure tines.

Exposure Time	LC_50_ (IJs/mL)	95% Confidence Interval	Concentration (IJs/mL)	LT_50_ (h)	95% Confidence Interval
24 h	1139	380–3411.60	50	96.99	84.10–111.90
48 h	173.40	128.89–233	100	84.31	74.10–95.96
72 h	94.57	75.20–119.30	150	66.81	59.40–75.14
96 h	69.18	53.54–89.40	200	49.29	44.20–54.97
120 h	57.67	44.40–74.90	250	45.03	40.60–49.96

**Table 4:** Toxicity of LZ-G7 bacteria on RPW adults after feeding with different concentrations.

Time	LC_50_ (CFU/mL)	95% Confidence Interval	Concentration (CFU/mL)	LT_50_ (h)	95% Confidence Interval
6 h	185.00 × 10^9^	1.95 × 10^8^–1.74 × 10^14^	0.01 × 10^7^	186.13	79.18–437.6
12 h	18.000 × 10^9^	2.83 × 10^8^–1.14 × 10^12^	0.10 × 10^7^	181.29	40.48–811.9
24 h	9.9500 × 10^9^	3.63 × 10^8^–2.72 × 10^11^	1.00 × 10^7^	166.52	68.91–402.4
36 h	1.7000 × 10^9^	1.64 × 10^8^–1.75 × 10^10^	5.00 × 10^7^	103.27	54.45–195.8
48 h	0.0457 × 10^9^	1.90 × 10^7^–1.10 × 10^8^	10.0 × 10^7^	37.450	29.80–47.07

**Table 5:** Toxicity of LZ-G7 bacteria on RPW larvae after feeding with different concentrations.

Time	LC_50_ (CFU/g)	95% Confidence Interval	Concentration (CFU/mL)	LT_50_ (h)	95% Confidence Interval
24 h	0.99 × 10^8^	0.43 × 10^8^–2.25 × 10^8^	0.10 × 10^8^	206.77	139.80–305.60
48 h	0.34 × 10^8^	0.28 × 10^8^–0.41 × 10^8^	0.15 × 10^8^	116.37	97.10–139.50
72 h	0.24 × 10^8^	0.21 × 10^8^–0.27 × 10^8^	0.20 × 10^8^	100.84	86.13–118.10
96 h	0.18 × 10^8^	0.17 × 10^8^–0.20 × 10^8^	0.25 × 10^8^	83.96	73.81–95.50
120 h	0.15 × 10^8^	0.14 × 10^8^–0.17 × 10^8^	0.30 × 10^8^	48.48	43.37–54.18

**Table 6:** Toxicity of LZ-G7 bacteria on RPW larvae after injection with different concentrations.

Time	LD_50_ (CFU)	95% Confidence Interval	Amount of Injected Bacteria (CFU)	LT_50_ (h)	95% Confidence Interval
6 h	76.58 × 10^6^	13.70 × 10^6^–427.90 × 10^6^	0.5 × 10^6^	59.29	42.32–83.05
12 h	7.77 × 10^6^	5.11 × 10^6^–11.81 × 10^6^	2 × 10^6^	29.37	23.73–36.36
24 h	3.03 × 10^6^	2.38 × 10^6^–3.86 × 10^6^	4 × 10^6^	19.41	16.57–22.73
36 h	1.34 × 10^6^	0.96 × 10^6^–1.87 × 10^6^	6 × 10^6^	16.89	14.69–19.41
48 h	0.63 × 10^6^	0.40 × 10^6^–1.00 × 10^6^	8 × 10^6^	11.10	09.32–13.22

## Data Availability

Data will be made available on request.
